# Design for Additive Bio-Manufacturing: From Patient-Specific Medical Devices to Rationally Designed Meta-Biomaterials

**DOI:** 10.3390/ijms18081607

**Published:** 2017-07-25

**Authors:** Amir A. Zadpoor

**Affiliations:** Additive Manufacturing Laboratory, Department of Biomechanical Engineering, Delft University of Technology (TU Delft), Mekelweg 2, 2628 CD Delft, The Netherlands; a.a.zadpoor@tudelft.nl; Tel.: +31-15-2781021; Fax: +31-15-2784717

**Keywords:** additive bio-manufacturing, design methodology, medical devices, meta-biomaterials, rational design, image-based design

## Abstract

Recent advances in additive manufacturing (AM) techniques in terms of accuracy, reliability, the range of processable materials, and commercial availability have made them promising candidates for production of functional parts including those used in the biomedical industry. The complexity-for-free feature offered by AM means that very complex designs become feasible to manufacture, while batch-size-indifference enables fabrication of fully patient-specific medical devices. Design for AM (DfAM) approaches aim to fully utilize those features for development of medical devices with substantially enhanced performance and biomaterials with unprecedented combinations of favorable properties that originate from complex geometrical designs at the micro-scale. This paper reviews the most important approaches in DfAM particularly those applicable to additive bio-manufacturing including image-based design pipelines, parametric and non-parametric designs, metamaterials, rational and computationally enabled design, topology optimization, and bio-inspired design. Areas with limited research have been identified and suggestions have been made for future research. The paper concludes with a brief discussion on the practical aspects of DfAM and the potential of combining AM with subtractive and formative manufacturing processes in so-called hybrid manufacturing processes.

## 1. Introduction

Additive manufacturing (AM) or, as it is sometimes called, 3D printing technologies have made tremendous progress during the last decade. The accuracy, repeatability, and reliability of AM-based production techniques have improved, as are the range of available materials. Optimizations of the processes for maximizing the manufacturing efficiency, minimizing the need for post-manufacturing steps, and the economy of scale have all contributed towards decreasing the costs associated with AM. As a result, AM has emerged as a viable option for fabrication of functional parts particularly in the high added value sectors of industry such as healthcare [1] and aerospace [2]. Given the possibilities offered by AM and their implications for the design process, design for AM (DfAM) has emerged as an important research topic during the last few years.

**Figure 1:**
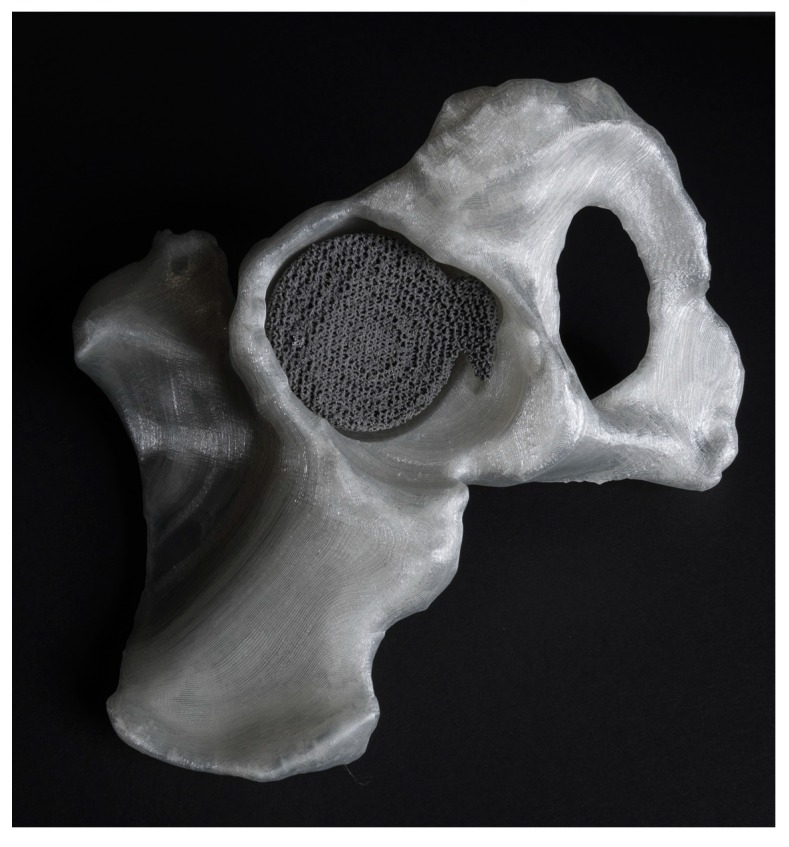
A patient-specific implant designed based on computed-tomography (CT) images and AM with selective laser melting. The corresponding bone is also segmented from CT images and AM using with fused deposition modeling. Made at Additive Manufacturing Lab, TU Delft (Researchers: Eline Kolken and Saber Amin Yavari).

In AM, complexity is free or at least very inexpensive as compared to conventional manufacturing techniques. There are also complex shapes that could only be manufactured using AM. It is therefore both technically possible and economically feasible to fabricate topologically complex designs with advanced functionalities that originate, at least partially, from topology. Furthermore, the form-freedom and complexity-for-free features offered by AM enable application of design optimization approaches that aim at minimizing weight, maximizing stiffness, improving fatigue life, and/or enhancing the longevity of medical implants. Finally, the design of every individual part of AM production batches could be different at no additional cost (batch-size-indifference). Tailor-made solutions with a continuous range of dimensions and infinite number of design variations that perfectly match the desired specifications therefore become possible. In the case of medical devices, that translates to “patient-specific” implants or instruments that perfectly match the anatomy of the patient (Figure 1). However, tailor-made solutions are not limited to the medical industry and are applicable in other sectors as well.

A wide range of AM technologies have been developed during the last few decades. The standard terminology of AM according to ISO/ASTM 52900 [3] defines seven categories of AM technologies. Not all categories of AM technologies are equally used for the fabrication of medical devices. The powder bed fusion processes are of particular interest in this regard, especially for metallic medical devices. Both selective laser melting (SLM) and electron beam melting (EBM), often used for manufacturing of metallic medical devices, are among the powder bed fusion processes. The powder bed fusion processes including selective laser sintering are also used for polymeric biomaterials. Another category of AM technologies that is often used in connection with medical devices is material extrusion. Fused deposition modeling (FDM) is perhaps the most widely used material extrusion technique that has found many applications in the medical industry. Vat polymerization techniques such as stereolithography are also used for manufacturing of polymeric biomaterials and medical devices. A more comprehensive review of the AM techniques that are usually used for fabrication of the different types of medically relevant materials and devices could be found in [1].

In this paper, we review all above-mentioned aspects of design for AM. Although the discussion is general in essence and applies to multiple sectors of the industry, medical devices and biomaterials will receive special attention.

## 2. Patient-Specific Designs

As opposed to conventional production techniques, varying the design from one AM production specimen to the next does not result in substantial cost increase, rendering tailor-made designs feasible. Tailor-made designs have multiple favorable features including the ability to personalize the product. In the context of medical devices [4], the term patient-specific refers to that personalization concept. Surgical instruments and guides [5,6], medical implants [7,8,9,10], prosthetics [11,12,13], and orthotics [14,15,16,17] could all be designed using patient-specific approaches and fabricated with AM. AM drugs where the dosage and the shape of the “oral dosage forms” could be personalized have recently received a lot of attention too [18,19,20,21]. Veterinary applications of some of the above-mentioned solutions have been also envisioned [22,23].

Personalization of AM products goes beyond the medical industry and is also applicable to customer products particularly the ones in which proper ergonomics is important [24,25,26]. A case for personalized AM products could be even made in the fashion [27,28,29] and food [30,31] industries. In the fashion industry, both the functional and aesthetics aspects of personalization are relevant. As for the functional aspect, AM clothes could be “tailor-made” to perfectly fit the intended customer. On the other hand, the aesthetics of fashion pieces could be personalized to utilize the added value of “uniqueness”. Moreover, the improved aesthetics of parts made with AM could be used also in the medical industry. For example, AM could be used to fabricate more aesthetically appealing prosthetics devices. Since “enhanced cosmesis” contributes towards the psychological well-being of the patients receiving prosthetic devices [32], better aesthetics achieved through AM may play a role in better acceptance of such devices by patients.

### 2.1. Image-Based Design Personalization

To personalize a specific design, one needs to acquire data regarding the geometry of certain parts of the human body, hence the need for imaging modalities. The design of ergonomic products, fashion pieces, and even most prosthetic and orthotic devices only requires information regarding the external geometrical features of the human body and not the shape of internal organs. For such applications, it is sufficient to use techniques such 3D scanning, which are nowadays inexpensive and widely available. Ordinary digital cameras including those found on smartphones, in combination with dedicated software, could also be used to obtain the three-dimensional shape of objects from a series of photos [33,34].

The patient-specific design of multiple types of medical devices including implants and (surgical) guides/instruments requires the shape of internal body organs that are usually acquired through medical imaging modalities such as computed tomography (CT) or magnetic resonance imaging (MRI). Both CT and MRI scanning yield a stack of two-dimensional images that include all the body parts within the field of view. Creating a three-dimensional computer model of a specific part of the body from such two-dimensional stack of images requires following a number of image processing steps. Dedicated medical image processing software has been developed for this (and similar) purposes. Examples of non-commercial software include imageJ [35,36] and DeVIDE [37], while examples of commercial software include Materialise Mimics, Amira, and Simpleware.

The first step is called image segmentation and aims at separating the body part of interest from other tissues/organs present in every single two-dimensional image of the image stack. Automated and semi-automated techniques have been developed for this purpose and are available in most above-mentioned software, where certain features of images are used for distinguishing different types of tissues from each other. For example, thresholding the gray values in CT images enables separation of the bony tissue that has higher (mineral) density and, thus, higher x-ray attenuation coefficient from other types of less dense tissues [38,39,40]. Once the contours of the desired body parts are detected in all images of the stack, the three-dimensional shape is obtained by connecting (and potentially interpolating between) those contours. The resulting three-dimensional model could then be represented in different formats including the STL (STereoLithography) format and exported to design software. Some of the above-mentioned medical image processing software packages have associated design software packages that enable seamless data transfer between both types of software and streamlined design experience. The process of creating three-dimensional models from a stack of CT or MRI images could be time consuming, laborious, and expensive.

**Figure 2:**
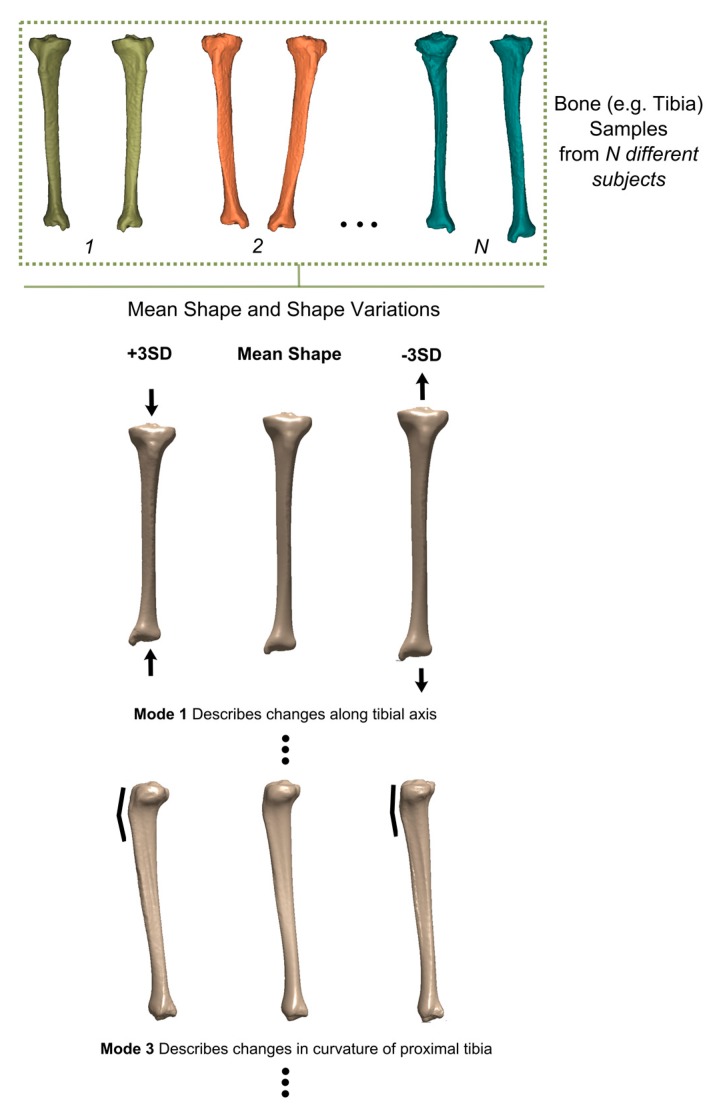
An example of a statistical shape model developed for tibia. Many instances of the bone shape (depicted with different colors) are used to make a mathematical representation of the mean bone shape and the main modes of shape variation from the mean shape. The model has been developed at TU Delft (Researchers: Nazli Tümer and Vahid Arbabi).

An important exception to the above-mentioned scenario is when a part or all of the native geometry is unavailable for imaging, for example, when a patient has lost a large part of their bone in a traumatic event such as a car accident or a sport-related injury. Another important example is when a tumor has developed in the body and has disfigured the original anatomical shape of the organ. In some cases, it is possible to simply use the contralateral side for estimating the anatomy of the disfigured or traumatized organ. In some other cases where the trauma is bilateral or too large (e.g., in acetabulum), no information regarding the native anatomy may be available. In such cases, it is possible to use the concept of “statistical shape models” [41,42] to estimate the geometry of the missing or disfigured organ based on the geometry of the remaining parts of the organ. Statistical shape models (SSM) [41,42] are templates of the body parts that are created based on the many instances of that body part found in the individuals of a specific population. A SSM describes the mean shape of that body part together with the main modes of variation from the mean shape in the context of a mathematical model (Figure 2). In the case where a part of the body part is missing in a patient, the SSM could be fitted to the remaining parts of the body part to estimate the shape of the missing parts.

### 2.2. Parametric and Non-Parametric Designs 

Once data regarding the geometry of the relevant body parts (either internal or external) are acquired, the designers need to tailor their design to the specific geometry at hand. This could be performed systematically through a parametric design approach where a number of parameters are used to personalize the design. A number of recent studies have proposed parametric design and modeling approaches that could eventually lead to robust paradigms for the parametric design of various medical devices [12,43,44]. At the core of many such approaches is the ability to describe the native anatomy of the patients using a mathematical model that requires a limited number of parameters. The mathematical model could then be used as a basis for parametric designs of medical devices.

Given the batch-size-indifference offered by AM, no specific relationships need to be used to link the various design parameters nor does the designer have to limit the values of the parameters to certain catalogued “sizes”, thereby allowing for truly personal designs. Moreover, it is at least in theory possible to automate the process of fitting the parametric design to the three-dimensional models of the body parts such as SSMs [42] or other types of mathematical models [43], meaning that the personalized design process could be at least partially automated. The systematic approach described in this section is, however, hampered by a few limitations of the existing technology. In particular, not so many parametric models are currently available for most (medical) applications partially due to the fact that geometrical complexities often make it difficult to develop parametric models that cover all types of anatomical variabilities. Most personalized designs particularly patient-specific designs are, thus, performed at least partially in a non-parametric way. Another advantage of the statistical shape models, described above, is that they enable more systematic development of parametric models. However, development of SSMs requires substantial expertise, time, and resources. Consequently, only limited number of SSMs are currently available for designers to work with.

## 3. Topologically Complex Designs and Metamaterials

The fact that topological complexity is for free in AM, liberates designers from many constraints that has traditionally limited the realistic reach of their imagination and, thus, the space of possible functionalities. The examples of places where complex topology could create or improve functionalities are numerous. However, metamaterials are perhaps the best platform within which the unusual properties originating from complex geometries could be studied. That is why, in this section, we will initially focus on metamaterials and will proceed to rational and computationally enabled design in the second sub-section.

### 3.1. Metamaterials

**Figure 3:**
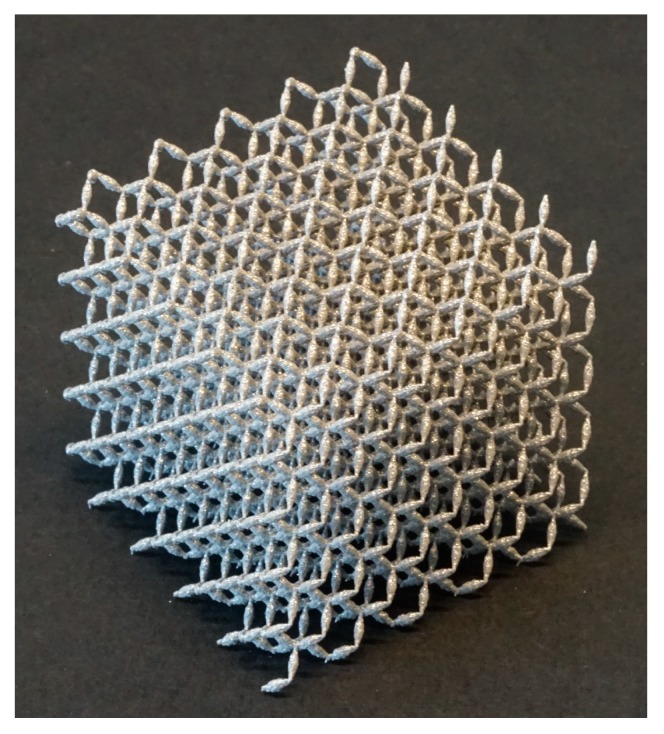
A penta-mode mechanical metamaterial made with selective laser melting at Additive Manufacturing Lab, TU Delft (Researchers: Reza Hedayati and Sander Leeflang) (specimen dimensions: 4 cm × 4 cm × 4 cm). The special geometry of the struts in this type of mechanical metamaterials results in some kind of fluid-like behavior. Moreover, the usual power-law relationship between the relative density and mechanical properties (Ashby’s laws) break down. For more details, see [69].

Metamaterials show certain sets of unusual physical properties at the large scale, which are not intrinsic to the materials they are made of, but are a consequence of the design and arrangement of their constituting components. The large-scale properties of metamaterials are, thus, a function of their small-scale topology [45,46]. Very complex micro-architectures may be needed to give rise to the desired sets of unusual physical properties, which is why design and manufacturing of metamaterials is intimately linked to developments in advanced AM techniques. Metamaterials are often named after the type of unusual physical properties one tries to achieve. Examples are optical metamaterials [47,48,49], acoustic metamaterials [50,51,52,53], mechanical metamaterials [54,55,56], and meta-biomaterials [57]. For example, mechanical metamaterials (see, e.g., Figure 3) with such unusual properties as negative Poisson’s ratio [58,59,60], negative elasticity [46], and negative compressibility [61,62,63] have been developed during the last few years. Such unusual properties have many applications in (soft) robotics [64,65], development of materials with advanced functionalities, flexible electronics [66], etc. Optical metamaterials, on the other hand, have been pursuing such ambitious targets as invisibility cloaks [67,68].

As for meta-biomaterials, certain combinations of mechanical, mass transport (e.g., permeability and diffusivity), and biological properties are often required for proper functioning of biomaterials. For example, biomaterials used for tissue regeneration usually need to provide enough mechanical support while having limited stiffness values so as to prevent the stress shielding phenomenon [70,71] that limits bone regeneration and might stimulate bone resorption. Ideally, one needs to have elastic properties in the range of those of the tissue that is replaced by the biomaterial, while having much higher values of mechanical strength.

**Figure 4:**
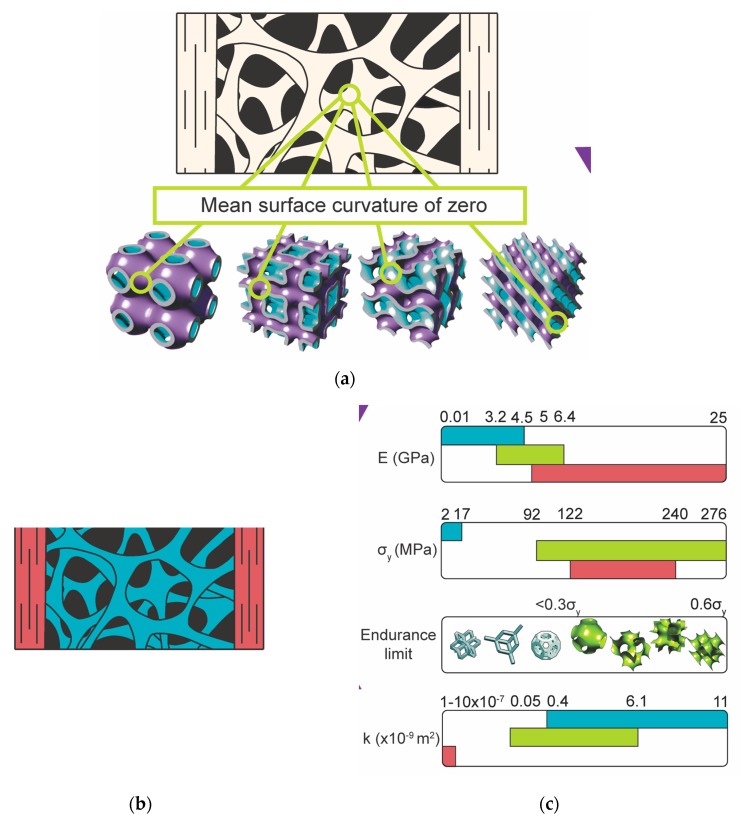
AM meta-biomaterials (**a**) based on triply periodic minimal surfaces that simultaneously mimic different types of bone (**b**) properties including topological, mechanical, and mass transport properties. They also show extremely long fatigue lives. The properties of these meta-biomaterials (light green) are compared with those of cortical (red) and trabecular (cyan) bone (**c**) (Researchers at TU Delft: Françoise Bobbert and Mohammad Ahmadi) (Reprinted from [75] with permission from Elsevier).

Natural materials usually cannot provide such combination of properties, because elastic modulus and strength are usually very tightly correlated with each other in natural materials. At the same time, there is a need for high permeability [72,73] and diffusivity [74] values of biomaterials to ensure enough cell nutrition and oxygenation during the earliest phases of tissue regeneration when blood vessels have not yet been re-generated. However, high permeability values require high levels of porosity, which reduce the mechanical properties of biomaterials. It is therefore very challenging to find natural materials or synthetic polymers that simultaneously satisfy all the requirements. Meta-biomaterials open an avenue where one could use the small-scale geometry to tune the properties of the material and find a solution that satisfies all the requirements. In a recent study, for example, we have shown that AM porous metallic biomaterials designed based on triply periodic minimal surfaces (Figure 4) could show the ideal combination of low elastic modulus in the high-end range of those observed for trabecular bone, high yield strengths that well exceed the strength of cortical bone, extremely long fatigue lives with endurance limits exceeding 60% of their yield strength, and permeability values in the range of those reported for natural human bone [75]. This kind of biomaterials could therefore mimic the properties of natural bone “to an unprecedented level of multi-physics detail” [75] and may open many new opportunities for development of high-preforming biomaterials.

In addition to providing a unique combination of properties, meta-biomaterials generally have much higher surface areas as compared to solid materials with corresponding shapes. This increase in the surface area (that could reach a few orders of magnitude in some cases) is due to the porous nature of most meta-biomaterials and could be used for amplifying the effects of surface bio-functionalization techniques to improve tissue regeneration performance [76] and prevent implant-associated infections [77].

### 3.2. Rational and Computationally Enabled Design

Given the fact that the properties of metamaterials are functions of their small-scale geometry, it is logical to use analytical and computational tools to rationally design metamaterials as well as other types of AM parts whose function and/or performance is related to their (topological) design. As for metamaterials, both analytical [78] and computational [79] techniques have been used to obtain topology–property relationships that could be used for adjusting the topology such that the resulting properties match the desired ones.

Obtaining this kind of topology–property relationships analytically is relatively straightforward when only one type of physical property is targeted and when the field equations governing the physics of the problem are linear and easy to solve. Important examples are the elastic properties of mechanical metamaterials or meta-biomaterials. Closed-form solutions that describe the relationship between the geometry and elastic properties of metamaterials have been obtained for different types of repeating unit cells particularly the ones based on beam-like structures [78]. Beam theories such as the Euler-Bernoulli and Timoshenko beam theories are often sufficient to describe the mechanical behavior of such meta-materials, rendering the process of obtaining analytical solutions relatively straightforward [78]. More advanced plate/shell theories may be needed for metamaterials based on shell-like structures such as the above-mentioned meta-biomaterials [75] that are based on triply periodic minimal surfaces. One advantage of obtaining closed-form topology–property relationships is that the process of rational design becomes relatively straightforward: one simply has to solve the closed-form topology–property equation for deciding what kind of geometry is needed to obtain the desired properties.

In many cases, however, it is realistically not possible to solve the governing equations analytically and computational models are needed for that purpose. A notable example is soft mechanical metamaterials that experience large deformations, instability, and buckling [80,81]. The nonlinear equations describing the deformation of such metamaterials are challenging to solve analytically, which highlights the importance of computational models. The need for computational models becomes clearer when considering the design problems where multiple types of physical properties need to be simultaneously taken into account such as the case of meta-biomaterials.

Another important aspect in the design of meta-biomaterials is the accuracy of the mechanical loads used for computational analysis of the mechanical behavior. Accurate estimation of the mechanical loads sustained by some types of meta-biomaterials such as (the fatigue behavior of) bone-mimicking biomaterials is not possible without computational models that could analyze musculoskeletal movements and the resulting musculoskeletal loads. It is therefore necessary to use (patient-specific) musculoskeletal models [82,83,84] or at least simpler versions of such models based on mass-spring-damper models [85,86,87] to obtain a realistic picture of what the actual mechanical loads experienced by AM meta-biomaterials are. Since the mechanical behavior of materials particularly their fatigue behavior is strongly dependent on the type of mechanical loading, such models could be useful in giving a more realistic prediction of the performance of meta-biomaterials in their actual service conditions. Combining models that are used for the rational design of different kind of metamaterials with detailed computational models that predict the mechanical loads experienced by those metamaterials has not been widely reported in the literature so far and is suggested as one of the areas for future research.

Finally, rational design for AM may require taking some aspects of the AM process into consideration. Most models used for the design of metamaterials as well as other types of AM parts do not take the manufacturing artifacts into accounts nor do they explicitly implement the manufacturing limitations in the design process. Some previous research has shown that the characteristic manufacturing irregularities associated with AM techniques could significantly reduce the mechanical properties of AM porous biomaterials [88]. It is therefore important to consider such aspects of the AM processes themselves when designing metamaterials and other parts that need to be ultimately fabricated using AM. Of course, manufacturing imperfections and geometrical irregularities are not unique to AM processes and have been reported to decrease the workability limits and mechanical properties in other types of manufacturing processes such as machined parts [89], plastically deformed sheet metals [90,91], and adhesively bonded constructs [92].

The models used for the rational design of metamaterials and AM parts should, thus, be coupled with the computational models developed for (optimizing) the AM processes [93,94,95]. The outputs of the process models in terms of surface roughness, thermal history, and residual stresses could then be introduced to the rational design models to improve their accuracy and representativeness. Once more, not much research has been done in this potentially fruitful direction of scientific inquiry.

## 4. Optimal and Bio-Inspired Designs

The possibility to fabricate geometrically complex structures at different scales as well as the ease of spatially distributing multiple materials enable new design routes that have been either difficult or impossible to realize with conventional manufacturing techniques. Two specific design routes have been highlighted in this section: topology optimization and bio-inspired design of functional (bio-)materials.

### 4.1. Topology Optimization

Topology optimization techniques [96] have been under development for a few decades. The aim of such algorithms is to optimally distribute the material within a structure such that certain objective functions are optimized. A classic problem for topology optimization algorithms is to optimally distribute a given amount of mass within a specific geometrical region such that the compliance of the structure is minimized (i.e., the stiffness is maximized). The objective function is therefore the compliance of the structure while the total mass may be implemented as an optimization constraint. Many algorithms have been already developed for solving such optimization problems. Prior to recent advances made in AM, engineers were limited to either subtractive (e.g., machining) or formative (casting, forming) manufacturing processes when trying to fabricate parts designed through topology optimization. The inherent limitations of both conventional routes of manufacturing were particularly problematic for the geometries resulting from topology optimization algorithms, because they tend to be irregular, include hollow enclosures, and be topologically rich. That is why the form-freedom offered by AM is such a welcome opportunity for application of the topology optimization algorithms. It is, moreover, possible to combine AM techniques with subtractive techniques not only for finishing the fabricated parts but also to expand the space of achievable topologies. Hybrid manufacturing processes that combine AM with subtractive (and perhaps formative) manufacturing are therefore expected to be interesting areas for future research, as more hybrid manufacturing machines become available.

Even though AM has very few limitations in terms of the geometries it could achieve, there are still some limitations. In particular, some AM techniques cannot fabricate geometries that include overhangs without using support structures. Supports structures are generally undesired, as they increase the manufacturing time, add an additional step for their removal to the fabrication process, and may result in decreased surface quality. It is therefore important to include additional constraints in the topology optimization algorithms to limit the number of overhangs. A number of such topology optimization algorithms have been proposed during the recent years (e.g., [97,98]).

**Figure 5:**
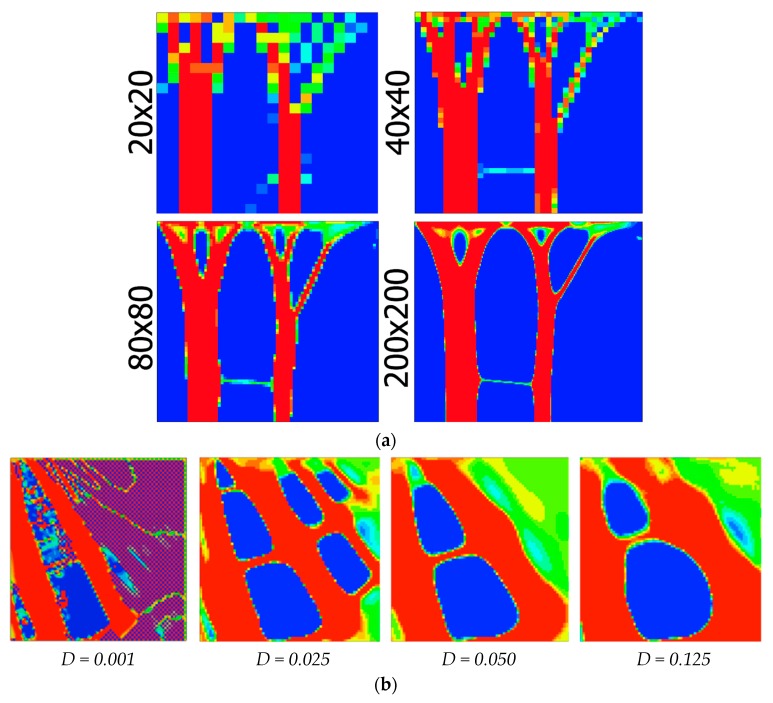
Bone tissue adaptation models working on the basis of homeostasis and driven by the strain energy density were implemented in finite element models to determine the distribution of density and, thus, mechanical properties under different loading conditions. The patterns emerging from such bone tissue adaptation algorithms (red = high bone density, blue = low bone density) are similar in shape and function to those resulting from topology optimization algorithms and could be used both for the general design of AM parts as well as for the patient-specific design of micro-architecture in AM porous implants (**a**). The level of detail could be adjusted using a coefficient, *D*, that regulates the size of the influence domain of the osteocytes (**b**) (Researcher at TU Delft: Gianni Campoli) (Reprinted from [104] with permission from Elsevier).

Topology optimization algorithms are in a way similar to the bone tissue adaptation process [99,100]. In both cases, the overall aim is to achieve adequate mechanical performance without making the structure overly massive. Indeed, some theories proposed for simulating the bone tissue adaptation are based on the assumption that this biological process basically works as a (global) optimization algorithm [101,102]. Although it is not clear what the best theoretical model for simulating bone tissue adaptation is, the analogy between bone tissue adaptation models and topology optimization algorithms have certain practical implications for the design of meta-biomaterials and implants. To design the small-scale geometry of meta-biomaterials as well as orthopedic implants, one could use either bone tissue adaptation models (Figure 5) or topology optimization algorithms. This takes the rational design one step further, because the bone-mimicking meta-biomaterial or implant is not being designed in generic terms but for a specific mechanical loading condition that corresponds to a specific patient. Although a number of studies have used computational models for the (rational) design of the micro-architecture of AM porous biomaterials [103], they are mostly limited to simple loading cases and usually do not include patient-specific aspects. It is therefore suggested that future studies use topology optimization algorithms and/or bone tissue adaptation models for patient-specific rational design of the micro-architecture of meta-biomaterials and (porous) orthopedic implants.

Similar to meta-biomaterials, other types of metamaterials could be also designed using topology optimization algorithms. For example, some studies have used topology optimization for the design of auxetic structures (i.e., structures with negative Poisson’s ratios).

### 4.2. Bio-Inspired Design

Nature uses very clever approaches to solve important design problems that seem impossible to solve using traditional approaches of design and manufacturing. Two important examples are presented here to illustrate the approaches used by nature for two problems that are also relevant for the design of advanced (bio-)materials and medical devices. The first problem refers to developing materials that simultaneously show high strength/stiffness and toughness. Most engineering materials show either high strength values or high toughness values [105]. Attempts to increase one usually result in lower values for the other. In comparison, a number of natural biomaterials such as bone, nacre, and spider silk show high values of both specific strength/stiffness and toughness [106], while being lightweight.

Another problem is the interface between extremely hard and extremely soft materials. Stress values rise to high levels at the interface between two materials with large stiffness disparity. Attachment of soft and hard materials is therefore very challenging, as tears and failure often occur at the interface of both materials. Materials with high levels of disparity in mechanical properties also exist in nature. Important examples are the interface between bone and cartilage or that of bone and tendon. As opposed to engineered soft-hard interfaces, the interfaces between hard and soft tissues rarely fail in nature. For example, failure and fatigue is often observed either in bone or in tendon but rarely at their interface [107].

To achieve such extraordinary combinations of mechanical properties as well as to bond extremely hard and extremely soft materials, nature uses very specific design approaches that are based on hierarchical structures, gradients in mechanical properties, and composite materials (combination of materials with different properties) [107,108,109,110]. Using those design strategies is not possible without access to advanced AM techniques that enable fabrication of complex multi-scale structures from multiple materials whose spatial distribution could be independently and precisely controlled. That is why AM techniques in general and multi-material AM in particular enable us to use bio-inspired approaches for solving some of the most complex material design problems [108]. Since such AM techniques have been only recently developed, only limited research is available on their application for realizing bio-inspired design approaches. More research is therefore needed in this direction.

## 5. Discussion

In the previous sections, we reviewed the differences between DfAM [111,112] and design for manufacturing (DFM). We discussed the different design approaches both in the context of general engineering practice as well as in the specific case of fabricating medical devices, biomaterials, and other biomedically relevant products. In summary, DFM aims to design products such that they are easy to manufacture, keeping the limitations of the conventional manufacturing processes in mind. In the case of AM, however, the ease and cost of manufacturing have little to do with the geometrical complexity of the design or the size of the production batch. The design objectives are therefore different in DfAM.

On the high end of medical devices and biomaterials, the design objective usually is to use the complexity-for-free and batch-size-indifference features to their fullest extents, thereby achieving products with superior or unprecedented functionalities. Concepts such as patient-specific medical devices including patient-specific implants, medical instruments, prosthetics and orthotics as well as meta-biomaterials are consistent with such a design paradigm. However, there is also something to be gained in the design of low-end medical devices and biomaterials. On-demand fabrication of medical devices in extreme conditions such as in war-hit zones or areas with insufficient access to healthcare products is an example.

There are also some design objectives that are important for both low-end and high-end applications. Designing non-assembly products and mechanisms is one such example. The ability to design and manufacture non-assembly medical devices is very appealing in extreme conditions similar to the ones mentioned above, as the skills required for the assembly of complex devices such as patient-specific prosthetics may be unavailable. On the other hand, non-assembly mechanisms with advanced functionalities and short lead times could offer many advantages in the high-end settings where multi-material AM techniques are available.

Combining AM with subtractive and formative processes could further enrich the space of achievable designs. In particular, combining additive and subtractive processes could lead to better control of the tolerances and surface finish as well shorter production times. Hybrid machines that combine AM and subtractive processes are currently under development [113,114]. Combining AM processes with formative processes, for example, through AM of negatives that could be later used as models are particularly interesting for a number of biomedical applications [115]. Moreover, the advantages of AM (e.g., form-freedom) could be combined with those of formative processes (e.g., a wide range of materials to work with).

From the cost-effectiveness viewpoint, the use of AM techniques could be justified as long as there are clear clinical benefits for the patients and clinicians. That is partially due to the fact that the costs associated with the manufacturing processes are relatively small as compared to the total cost particularly in treatment of challenging clinical cases. In some extreme cases such as the management of recurring and complex implant-associated infections, the cost of the material and manufacturing may be as little as 1% of the total cost associated with the management of infection episodes. That is why even a relatively modest improvement in the implant performance through an improved manufacturing process may be justifiable in terms of the associated costs. The costs associated with the DfAM process are, however, another story. Most of the steps required for designing medical devices and biomaterials are currently performed manually and are, thus, very expensive. For example, the largest fraction of the cost of AM patient-specific medical devices are those associated with the design and not the manufacturing process. That highlights the importance of automating and streamlining the process of designing patient-specific medical devices as well as other DfAM processes discussed here. That would allow more patients to benefit from what AM and DfAM have to offer in terms of improving the healthcare quality.

The technologies that are required for additive bio-manufacturing including both AM and imaging techniques have a number of limitations. As for imaging, the resolution and quality of medical images is often limited partly because high resolution images require longer scanning times, which result in higher doses of ionizing radiation as well as higher costs. Future developments in imaging modalities and protocol design (e.g., [116,117,118]) could help in addressing some of those limitations, thereby delivering higher resolution images that could then result in improved accuracy of the patient-specific design processes. The most important limitations of the AM process itself are the limited number of available materials as well as insufficient accuracy for some applications. The materials used for additive bio-manufacturing should be always biocompatible and for certain applications also biodegradable [119,120]. There are currently only a limited number of materials that could satisfy those requirements particularly given the strict biocompatibility requirements internally implantable medical devices. Future developments in AM techniques and availability of new biomaterials are required to address those limitations.

## 6. Conclusions

AM offers unparalleled levels of freedom in the design of medical devices and biomaterials. The principal aim of design for AM is to optimally use the opportunities offered by AM to achieve enhanced levels of medical device performance as well as biomaterials with unprecedented properties and, thus, functionalities. While imaging-based techniques are the most important tools for patient-specific design of medical devices, rational design approaches including those based on computational modeling and topology optimization seem to be the best suited pathways for development of biomaterials with unusually favorable sets of properties (i.e., meta-biomaterials). In addition, advanced (multi-material) AM techniques have enabled the application of bio-inspired principles in the design of medical devices and biomaterials. Many design problems are, however, still open and need to be addressed using the above-mentioned methodologies.
